# “Transition to CAMHS SPR” – a Simulation Induction Course Provided for Newly Appointed Child and Adolescent Mental Health Service (CAMHS) Higher Trainees (HTs)

**DOI:** 10.1192/bjo.2022.159

**Published:** 2022-06-20

**Authors:** Claire Tiley, Alina Cuhraja, Aleks Saunders, Kiran Virk, Marcela Schilderman, Emma Baxey, Megan Fisher

**Affiliations:** 1 Maudsley Learning, South London and Maudsley NHS Foundation Trust, London, United Kingdom; 2 Whittington Health NHS Trust, London, United Kingdom

## Abstract

**Aims:**

The transition between Core Psychiatry Training (CPT) and Psychiatry HTs is often anxiously anticipated by trainee psychiatrists, in view of the heightened responsibility and increased demand faced by trainees. The author wrote and delivered a one-day simulation induction course for newly appointed CAMHS HTs across London. The aim of this course was to improve participant's confidence, skills and knowledge in managing a range of conditions and challenging scenarios in children and young people (CYP) presenting to CAMHS. The course was also designed to improve HT's confidence in supporting junior colleagues and in managing conflict resolution. There was also an overarching aim of increasing human factor skills by focusing on these within the scenarios and debriefs.

**Methods:**

The simulation training was delivered online and consisted of five scenarios commonly faced by CAMHS SPRs based in a variety of settings. Themes within the scenarios included eating disorders and deliberate self-harm, as well as managing risk, multiple demands, and the psychosocial factors contributing to mental illness. Professional actors, plants and virtual backgrounds were used to enhance fidelity of the scenarios. Platform orientation and an introduction to simulation were initially provided followed by “ice breaker” activities, which were used to promote psychological safety amongst participants. Each scenario lasted approximately 10 minutes. Following each scenario, participants were supported to engage in a debrief using the Maudsley Debrief model. Pre- and post-course evaluation questionnaires were given to participants to complete and comparative analysis was conducted.

**Results:**

Seven participants completed both the pre- and post- course evaluation questionnaires. The mean sum score for course specific questions was 51.86 (SD = 9.56) pre course, and 68.00 (SD = 10.08) post course, showing a 31.12% increase in knowledge, skills, and confidence across the course specific domains.

The mean sum score for the Human Factors Skills for Healthcare Instrument (HFSHI) was 76.67 (SD = 17.26) pre course, and 86.50 (SD = 16.54) post course, showing a 12.82% increase in human factors skills.

**Conclusion:**

This simulation course demonstrated it is an effective and innovative way to help with induction for HT, resulting in an increase in knowledge, skills and confidence in trainees transitioning from CPT to HT, both in terms of factors specific to managing CYP's care and in relation to broader human factor skills.

